# Association between Immune-Related Adverse Events and Atezolizumab in Previously Treated Patients with Unresectable Advanced or Recurrent Non–Small Cell Lung Cancer

**DOI:** 10.1158/2767-9764.CRC-24-0212

**Published:** 2024-11-01

**Authors:** Hidetoshi Hayashi, Makoto Nishio, Hiroaki Akamatsu, Yasushi Goto, Satoru Miura, Akihiko Gemma, Ichiro Yoshino, Toshihiro Misumi, Takashi Kijima, Naoto Takase, Masaki Fujita, Sadatomo Tasaka, Atsuto Mouri, Tetsuro Kondo, Kei Takamura, Yosuke Kawashima, Kazuyoshi Imaizumi, Shunichiro Iwasawa, Shintaro Nakagawa, Tetsuya Mitsudomi

**Affiliations:** 1 Department of Medical Oncology, Faculty of Medicine, Kindai University, Osaka, Japan.; 2 Department of Thoracic Medical Oncology, The Cancer Institute Hospital of Japanese Foundation for Cancer Research, Tokyo, Japan.; 3 Internal Medicine III, Wakayama Medical University, Wakayama, Japan.; 4 Department of Thoracic Oncology, National Cancer Center Hospital, Tokyo, Japan.; 5 Department of Internal Medicine, Niigata Cancer Center Hospital, Niigata, Japan.; 6 Department of Pulmonary Medicine and Oncology, Nippon Medical School, Graduate School of Medicine, Tokyo, Japan.; 7 Department of General Thoracic Surgery, Chiba University Graduate School of Medicine, Chiba, Japan.; 8 Department of Biostatistics, Yokohama City University School of Medicine, Kanagawa, Japan.; 9 Department of Respiratory Medicine and Hematology, Hyogo Medical University, School of Medicine, Hyogo, Japan.; 10 Department of Medical Oncology, Takarazuka City Hospital, Hyogo, Japan.; 11 Department of Respiratory Medicine, Fukuoka University Hospital, Fukuoka, Japan.; 12 Department of Respiratory Medicine, Hirosaki University Graduate School of Medicine, Aomori, Japan.; 13 Department of Respiratory Medicine, International Medical Center, Saitama Medical University, Saitama, Japan.; 14 Department of Thoracic Oncology, Kanagawa Cancer Center, Kanagawa, Japan.; 15 Department of Respiratory Medicine, Obihiro-Kosei General Hospital, Hokkaido, Japan.; 16 Department of Pulmonary Medicine, Sendai Kousei Hospital, Miyagi, Japan.; 17 Department of Respiratory Medicine, Fujita Health University School of Medicine, Toyoake, Japan.; 18 Chugai Pharmaceutical Co., Ltd., Tokyo, Japan.; 19 Kindai Hospital Global Research Alliance Center and Thoracic Surgery, Kindai University, Osaka, Japan.

## Abstract

**Purpose:**

Real-world, large-scale studies on the association between immune-related adverse events (irAE) and immune checkpoint inhibitor therapy effectiveness are limited. We evaluated overall survival (OS) and progression-free survival based on the occurrence and grade of irAEs.

**Patients and Methods:**

We used data from Japanese patients with unresectable advanced or recurrent non–small cell lung cancer (NSCLC) who received atezolizumab and were enrolled in J-TAIL, a multicenter, prospective, single-arm observational study.

**Results:**

Among the 1,002 patients, 190 (19.0%) developed irAEs. The most common irAEs were skin disorders (3.8%) of any grade and interstitial lung disease (1.5%) of grade ≥3. Patients who developed irAEs within 4 or 6 weeks of treatment initiation had higher baseline C-reactive protein levels than those without irAEs. OS was longer in patients with irAEs [HR, 0.66; 95% confidence interval (CI), 0.54–0.82], particularly in those with low-grade irAEs (HR, 0.45; 95% CI, 0.33–0.62), than in patients without irAEs. The HR (95% CI) for OS in patients with low-grade and high-grade skin or endocrine disorder–related irAEs was 0.42 (0.28–0.64) and 0.37 (0.15–0.88), respectively. The HR (95% CI) for OS in patients with low-grade and high-grade irAEs other than skin or endocrine disorders was 0.44 (0.30–0.65) and 1.27 (0.96–1.69), respectively.

**Conclusions:**

In patients with unresectable advanced or recurrent NSCLC treated with atezolizumab in real-world settings, irAEs are associated with a clinical benefit except in those with high-grade irAEs other than skin and endocrine disorders.

**Significance:**

Immune checkpoint inhibitors are useful for treating NSCLC but can cause life-threatening irAEs. This study had a large sample size and stratified the analysis by irAE type and grade. The results suggest that improved management of irAEs may improve the therapeutic effect of atezolizumab.

## Introduction

Immune checkpoint inhibitors (ICI), such as antibodies against programmed cell death-1, PD-L1, or cytotoxic T lymphocyte–associated protein-4, are an evolving treatment option for non–small cell lung cancer (NSCLC; refs. 1–4). Treatment with ICIs is frequently accompanied by immune-related adverse events (irAE; refs. 5, 6). A systematic review of more than 5,000 patients with advanced NSCLC treated with ICIs revealed that the incidence of irAEs was 16% (3% grade ≥3) for anti–programmed cell death-1 agents and 11% (5% grade ≥3) for anti–PD-L1 agents (7). Although the use of ICIs has improved the therapeutic landscape for patients with NSCLC, not all patients benefit from ICI treatment and some experience life-threatening immunotoxicities (8). The effectiveness of ICIs depends on several factors, including PD-L1 expression in tumors, tumor mutation burden, T cell–inflamed tumor microenvironment, C-reactive protein (CRP) level, and the neutrophil-to-lymphocyte ratio (NLR; refs. 9–14).

irAEs have garnered considerable clinical and mechanistic interest and are thought to be primarily mediated by T cells (15). Patients who develop irAEs tend to have lower baseline cytokine levels and greater posttreatment increases in cytokine levels, suggesting an association between the underlying immune dysregulation and increased risk of irAEs (16). The development of irAEs is associated with survival benefit in melanoma (17–19) and NSCLC (20–25). However, the association with survival outcomes differs according to the type of irAE. Skin irAEs (scaly plaques and pruritus; refs. 24, 26–29) and immune-related thyroiditis (especially in patients with antithyroid antibodies; refs. 25, 27, 30–32) are thought to predict better outcomes in patients with NSCLC treated with ICIs, whereas pneumonitis is associated with worse survival (27, 33, 34). Diarrhea and increased liver enzyme levels do not seem to be associated with the effectiveness of ICIs (20). These reports suggest that appropriate management of irAEs is required to maximize the therapeutic effect of ICIs.

Although some studies have found an association between ICI therapy and irAEs (35–37), and the results of a pooled analysis of phase III randomized clinical trials examining the occurrence of irAEs and their effectiveness have been reported (38), only few large studies have been conducted in real-world clinical settings. In J-TAIL, a multicenter, prospective, single-arm observational study, the effectiveness and safety of atezolizumab monotherapy were evaluated in a large sample of Japanese patients with unresectable advanced or recurrent NSCLC in a real-world setting (39). In this study, we conducted a secondary analysis to examine the association between irAEs and clinical outcomes using large real-world clinical patient data.

## Materials and Methods

### Study design and treatment

The J-TAIL study was a multicenter, noninterventional, nonblinded, single-arm, prospective observational study (39). Consecutive patients scheduled to receive atezolizumab monotherapy were enrolled at 197 institutions in Japan from August 15, 2018, to October 16, 2019. Decisions about dose interruption or withdrawal of atezolizumab were made at the discretion of the treating physician in accordance with the atezolizumab package insert and guidelines for promoting optimal use. The J-TAIL study was conducted in accordance with the Declaration of Helsinki, Ethical Guidelines for Medical and Health Research Involving Human Subjects, and the International Council for Harmonisation guidelines for Good Clinical Practice. All patients provided written informed consent for study participation. The J-TAIL study was registered with UMIN Clinical Trials Registry under the identifier UMIN000033133 and with ClinicalTrials.gov under the identifier NCT03645330. This study was approved by the ethics committee at each hospital.

### Patients

Details of eligibility criteria have been reported previously (39). Patients scheduled to receive atezolizumab monotherapy who met the following enrollment criteria were included: ages ≥20 years at the time of providing consent, diagnosed as having unresectable advanced or recurrent NSCLC, and previously treated with systemic therapy. Patients were excluded if they were deemed unsuitable for participation by the investigator.

### Assessments

Progression and response were assessed according to the RECIST version 1.1 (40), without confirmatory measurement. Adverse event (AE) information, such as whether the event was an irAE, its severity, and its causal relationship to atezolizumab, was analyzed according to investigator assessment, consistent with the approach in previous studies (20–25). The severity of irAEs was assessed according to the NCI Common Terminology Criteria for Adverse Events version 4.0 (41), and the worst grade was used for analyses.

### Statistical methods

The analysis of irAEs and ICI effectiveness was prespecified in the protocol and statistical analysis plan. The safety analysis set included patients who received at least one dose of the study drug after enrollment and was used to assess irAEs, overall survival (OS), progression-free survival (PFS), and association between irAEs and ICI effectiveness.

Descriptive statistics were used to summarize patient baseline characteristics, using median (IQR or range) for continuous variables and *n* (%) for categorical variables. For OS and PFS, a Kaplan–Meier curve was constructed to calculate the median time-to-event, and 95% confidence intervals (CI) were calculated using the Brookmeyer–Crowley method. OS was defined as the time from the initiation of atezolizumab monotherapy to the date of death from any cause. Patients with no reported events, who did not die or were lost to follow-up, were censored on the last date they were known to be alive. PFS was defined as the time from the initiation of atezolizumab monotherapy to the date of the first documented progressive disease according to RECIST criteria, the date of clinical progression not based on imaging, or the date of death from any cause, whichever occurred first. Patients with no events reported, who did not progress, did not die, or were lost to follow-up, were censored on the date of their last evaluable tumor assessment. HRs and 95% CIs were calculated using the Cox proportional hazards regression, and comparisons between groups were performed using the log-rank test. Landmark analyses for OS and PFS were performed to avoid lead-time bias due to the time-dependent nature of irAEs and included all patients except those who experienced rapid progression. In the landmark analysis, patients who had no PFS events up to the landmark and who were followed up after the landmark at 4, 8, 12, and 24 weeks were included, and patients were classified according to whether they had any irAEs up to the landmark. Patients without an irAE were defined as those who did not experience any irAE throughout the entire follow-up period. Differences between groups of patients who developed irAEs and those who did not were tested using the Mann–Whitney *U* test for factors reported as predictors of ICI effectiveness, including PD-L1 expression, tumor volume, CRP level, and the NLR. To confirm the associations with OS or PFS and the onset of irAEs, multivariable Cox regression analyses were conducted. Variables included in the analyses were selected based on clinical relevance (sex, age [<75/≥75 years], Eastern Cooperative Oncology Group performance status, targetable driver oncogene status, previous treatment with ICIs, PD-L1 expression, and onset of irAEs). No imputation method was used for missing data. For all analyses, *P* values less than 0.05 were judged nominally significant without multiplicity adjustment. All statistical analyses were conducted using SAS version 9.4 (SAS Institute Inc.).

### Data availability

The datasets generated during and/or analyzed during the current study are available from the corresponding author on reasonable request.

## Results

### Patients

A total of 1,002 patients with unresectable advanced or recurrent NSCLC scheduled to receive atezolizumab monotherapy were included in the safety analysis. Among them, two patients were excluded from the analysis: one because of failure to appropriately obtain consent, and the other because atezolizumab was provided as the first-line treatment. The median follow-up was 11.5 months (IQR, 4.3–20.3).

The baseline demographic and clinical characteristics of patients included in the safety analysis and patients with and without irAEs are shown in Supplementary Table S1. Of the patients, 71.8% were men, and the median age was 71 years (range, 34–93 years). Among patients with and without irAEs, 30.5% and 28.7% were ages >75 years, 20.5% and 21.8% had squamous cell carcinoma, 7.9% and 12.9% had Eastern Cooperative Oncology Group performance status 2 or higher, and 11.1% and 5.8% had coexisting autoimmune disease, respectively.

### Incidence of irAEs

The frequency of irAEs of special interest by Common Terminology Criteria for Adverse Events grade is shown in Table 1. The most common irAE was skin disorder (3.8%), followed by interstitial lung disease (3.4%), thyroid dysfunction (2.9%), and liver dysfunction (1.6%) occurring in more than 1% of patients. The most common grade ≥3 irAEs was interstitial lung disease, with grade 3 interstitial lung disease occurring in nine patients (0.9%) and grade 5 interstitial lung disease occurring in six patients (0.6%).

**Table 1:** Frequency of irAEs of interest by grade in patients with NSCLC treated with atezolizumab

	Any grade	Grade 1	Grade 2	Grade 3	Grade 4	Grade 5
AE, *n* (%)	143 (14.3)	33 (3.3)	51 (5.1)	37 (3.7)	10 (1.0)	11 (1.1)
Skin disorders	38 (3.8)	17 (1.7)	15 (1.5)	6 (0.6)	0 (0)	0 (0)
Interstitial lung disease	34 (3.4)	7 (0.7)	12 (1.2)	9 (0.9)	0 (0)	6 (0.6)
Thyroid dysfunction	29 (2.9)	10 (1.0)	19 (1.9)	0 (0)	0 (0)	0 (0)
Liver dysfunction	16 (1.6)	4 (0.4)	4 (0.4)	5 (0.5)	2 (0.2)	1 (0.1)
Adrenal gland dysfunction	9 (0.9)	0 (0)	3 (0.3)	4 (0.4)	1 (0.1)	1 (0.1)
Encephalitis and meningitis	6 (0.6)	0 (0)	0 (0)	2 (0.2)	3 (0.3)	1 (0.1)
Severe diarrhea	5 (0.5)	2 (0.2)	3 (0.3)	0 (0)	0 (0)	0 (0)
Renal dysfunction	5 (0.5)	2 (0.2)	0 (0)	1 (0.1)	1 (0.1)	1 (0.1)
Colitis	5 (0.5)	2 (0.2)	1 (0.1)	2 (0.2)	0 (0)	0 (0)
Neuropathy	4 (0.4)	0 (0)	1 (0.1)	3 (0.3)	0 (0)	0 (0)
Myositis^a^	3 (0.3)	0 (0)	0 (0)	2 (0.2)	0 (0)	0 (0)
Myasthenia gravis	3 (0.3)	0 (0)	0 (0)	3 (0.3)	0 (0)	0 (0)
Type 1 diabetes mellitus	2 (0.2)	0 (0)	0 (0)	0 (0)	2 (0.2)	0 (0)
Hemophagocytic syndrome	2 (0.2)	0 (0)	0 (0)	1 (0.1)	1 (0.1)	0 (0)
Myocarditis	2 (0.2)	1 (0.1)	0 (0)	0 (0)	0 (0)	1 (0.1)
Rhabdomyolysis	1 (0.1)	0 (0)	0 (0)	1 (0.1)	0 (0)	0 (0)
Pituitary dysfunction	1 (0.1)	0 (0)	0 (0)	0 (0)	1 (0.1)	0 (0)
Hemolytic anemia	1 (0.1)	0 (0)	0 (0)	1 (0.1)	0 (0)	0 (0)

The severity of irAEs was assessed according to the NCI Common Terminology Criteria for Adverse Events version 4.0.

a There was one event with undetermined grade.

### Association between the occurrence of irAEs and the effectiveness of atezolizumab

**Figure 1:**
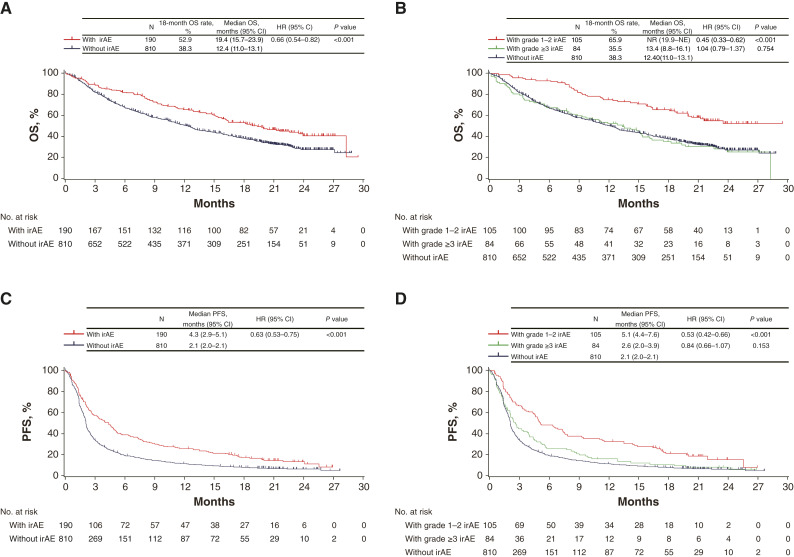
Association between the occurrences of irAEs and OS or PFS. **A,** OS according to the onset of irAE, (**B**) OS according to the grade of irAE, (**C**) PFS according to the onset of irAE, and (**D**) PFS according to the grade of irAE. One event with undetermined grade was excluded from the analysis according to grade. NE, not evaluable; NR, not reached.

Figure 1 shows OS and PFS according to the occurrence and grade of irAEs, regardless of the type. Patients with irAEs had longer OS than those without irAEs (HR, 0.66; 95% CI, 0.54–0.82; log-rank *P* < 0.001; Fig. 1A). The OS of patients with grade ≥3 irAEs was comparable to that of patients without irAEs (HR, 1.04; 95% CI, 0.79–1.37; log-rank *P* = 0.754), whereas patients with grade 1 to 2 irAEs had a longer OS (HR, 0.45; 95% CI, 0.33–0.62; log-rank *P* < 0.001; Fig. 1B), indicating that the OS benefit for patients with irAEs was primarily derived from low-grade irAEs. The trend of PFS was comparable to that of OS (Fig. 1C and D). Multivariable analyses showed that the onset of irAEs was an independent risk factor for both OS and PFS (Supplementary Tables S2 and S3).

**Figure 2:**
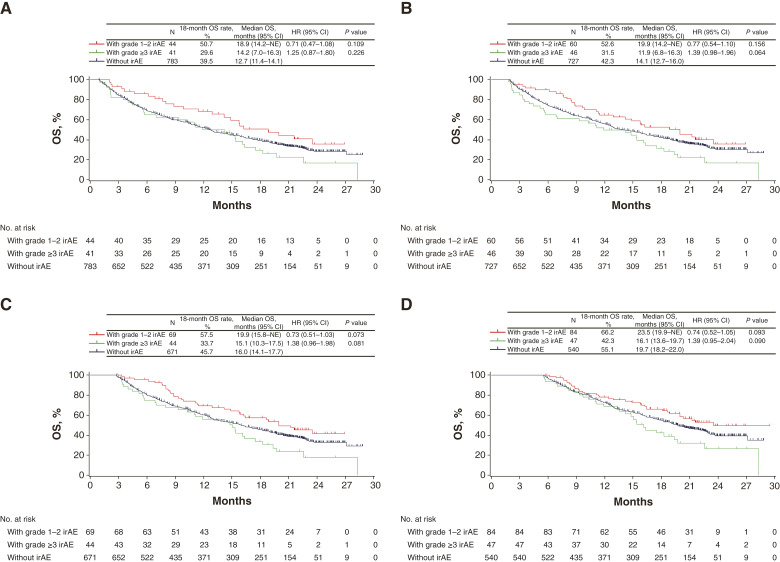
Landmark analysis of OS according to the grade of irAE. **A,** Four-week landmark OS according to the grade of irAE, (**B**) 8-week landmark OS according to the grade of irAE, (**C**) 12-week landmark OS according to the grade of irAE, and (**D**) 24-week landmark OS according to the grade of irAE. One event with an undetermined grade was excluded from the analysis. NE, not evaluable.

It is possible that if a patient is treated with atezolizumab for a long time, the total dose will be greater and the observation period will be longer, which may lead to a higher incidence of irAEs. To avoid this bias, we also performed a landmark analysis of OS. The HRs in patients with grade 1 to 2 irAEs were 0.71 (95% CI, 0.47–1.08; log-rank *P* = 0.109) for the 4-week, 0.77 (95% CI, 0.54–1.10; log-rank *P* = 0.156) for the 8-week, 0.73 (95% CI, 0.51–1.03; log-rank *P* = 0.073) for the 12-week, and 0.74 (95% CI, 0.52–1.05; log-rank *P* = 0.093) for the 24-week landmarks; the HRs in those with grade ≥3 irAEs were 1.25 (95% CI, 0.87–1.80; log-rank *P* = 0.226) for the 4-week, 1.39 (95% CI, 0.98–1.96; log-rank *P* = 0.064) for the 8-week, 1.38 (95% CI, 0.96–1.98; log-rank *P* = 0.081) for the 12-week, and 1.39 (95% CI, 0.95–2.04; log-rank *P* = 0.090) for the 24-week landmarks (Fig. 2A–D). Landmark analysis of PFS using 4 and 8 weeks as landmarks is depicted in Supplementary Fig. S1A and S1B.

**Figure 3:**
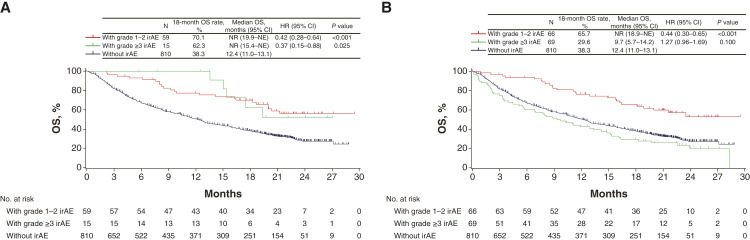
Association between the type of irAE and OS. **A,** OS according to the grade of irAE in skin or endocrine disorders and (**B**) OS according to the grade of irAEs affecting other systems (other than skin or endocrine disorders). One event with an undetermined grade was excluded from the analysis. NE, not evaluable; NR, not reached.

To explore the association between the occurrence of irAEs of different types and OS, we classified irAEs into two categories: skin or endocrine disorders, which are associated with improved survival in previous studies (Fig. 3A; refs. 24–32), and other types of irAEs (Fig. 3B). Compared with those for patients without irAEs, the HRs of OS were 0.42 (95% CI, 0.28–0.64; log-rank *P* < 0.001) for patients with grade 1 to 2 irAEs and 0.37 in those with high-grade irAEs (95% CI, 0.15–0.88; log-rank *P* = 0.025; Fig. 3A). With regard to irAEs other than skin or endocrine disorders, compared with those for patients without irAEs, the HRs of OS were 0.44 (95% CI, 0.30–0.65; log-rank *P* < 0.001) and 1.27 (95% CI, 0.96–1.69; log-rank *P* = 0.100; Fig. 3B), respectively. Overall PFS according to the type of irAE is shown in Supplementary Figs. S2A, S2B, S3A, and S3B.

In addition, 48 patients (4.8%) experienced multiple irAEs. These patients had longer OS than patients with only a single irAE (HR, 0.58; 95% CI, 0.35–0.95; log-rank *P* = 0.031; see Supplementary Fig. S4, which demonstrates the association between OS and the number of irAEs).

### Association between the occurrence of irAEs and predictors of ICI effectiveness

Table 2 shows the differences in predictors of the effectiveness of ICIs at baseline between patients with and without irAEs. No significant difference in PD-L1 expression was found between patients with and without irAEs. When irAEs that occurred during the entire observation period were considered, the NLR and tumor volume were lower in patients with irAEs than in those without irAEs (median NLR, 2.91 vs. 3.45; *P* = 0.005; median tumor volume, 42 vs. 47; *P* = 0.038). However, neither the NLR nor tumor volume was significantly different when limited to irAEs that occurred within 4 or 6 weeks after treatment initiation. No significant difference in CRP levels was observed in patients with and without irAEs when irAEs were considered for the entire observation period; however, when limited to irAEs within 4 or 6 weeks after the initiation of treatment, the CRP levels were higher in patients with irAEs than in those without irAEs (within the first 4 weeks: median CRP level, 0.90 vs. 0.42 mg/dL; *P* = 0.010; within the first 6 weeks: median CRP level, 0.66 vs. 0.38 mg/dL; *P* = 0.020). Results by grade and by type of irAE are shown in Supplementary Tables S4–S6.

**Table 2:** Association between ICI effectiveness predictors and irAEs in patients with NSCLC treated with atezolizumab

		Overall		Within the first 4 weeks		Within the first 6 weeks	
		With	Without	With	Without^b^	With	Without^b^
Characteristic		*N* = 190	*N* = 812	*N* = 82	*N* = 681	*N* = 77	*N* = 596
PD-L1 IHC (22C3), %	*N*	86	412	39	341	39	298
	Mean ± SD	22.7 ± 31.8	23.7 ± 31.4	21.7 ± 29.4	23.2 ± 31.2	21.2 ± 28.7	23.1 ± 31.6
	Median	5.0	5.0	5.0	5.0	5.0	5.0
	Q1, Q3	0.0, 30.0	0.0, 40.0	0.0, 30.0	0.0, 40.0	0.0, 30.0	0.0, 35.0
	Min, max	0, 95	0, 100	0, 90	0, 100	0, 90	0, 100
	*P* value^a^	0.915		0.721		0.737	
NLR	*N*	187	784	82	658	77	574
	Mean ± SD	4.07 ± 4.44	5.36 ± 7.62	3.96 ± 3.49	4.51 ± 5.21	3.61 ± 2.41	4.30 ± 5.06
	Median	2.91	3.45	2.92	3.17	2.91	3.02
	Q1, Q3	2.10, 4.60	2.24, 5.59	2.11, 4.62	2.12, 4.99	2.02, 4.62	2.07, 4.78
	Min, max	0.5, 33.0	0.6, 109.1	0.9, 26.6	0.6, 67.7	0.9, 14.2	0.6, 67.7
	*P* value^a^	**0.005**		0.540		0.498	
CRP, mg/dL	*N*	189	793	81	665	77	582
	Mean ± SD	1.69 ± 3.09	2.05 ± 3.69	1.96 ± 2.72	1.53 ± 2.82	1.78 ± 2.60	1.38 ± 2.54
	Median	0.51	0.58	0.90	0.42	0.66	0.38
	Q1, Q3	0.14, 1.83	0.15, 2.11	0.24, 2.20	0.13, 1.60	0.23, 1.86	0.12, 1.41
	Min, max	0.0, 26.3	0.0, 39.4	0.0, 11.8	0.0, 25.5	0.0, 11.8	0.0, 22.9
	*P* value^a^	0.560		**0.010**		**0.020**	
Tumor volume, mm	*N*	166	666	75	555	72	483
	Mean ± SD	48.88 ± 29.02	56.54 ± 36.98	54.01 ± 31.94	53.69 ± 34.32	48.86 ± 29.19	52.38 ± 33.99
	Median	42.00	47.00	45.00	45.00	42.35	44.00
	Q1, Q3	29.00, 62.00	30.00, 74.10	33.00, 63.50	29.00, 70.10	28.79, 61.40	28.00, 69.00
	Min, max	10.0, 161.0	10.0, 244.4	10.0, 149.0	10.0, 244.4	10.0, 149.0	10.0, 244.4
	*P* value^a^	**0.038**		0.685		0.595	

Abbreviation: Q, quartile.

a Mann–Whitney *U* test.

b Patients who had progression within 4 or 6 weeks after the initiation of treatment were excluded.

## Discussion

We conducted a secondary analysis to explore the association between irAEs and clinical outcomes using a large amount of real-world clinical patient data from the J-TAIL study and evaluated the effectiveness and safety of atezolizumab monotherapy in Japanese patients with unresectable advanced or recurrent NSCLC.

Consistent with previous studies (20–25, 36, 38), we found that the occurrence of irAEs, particularly low-grade irAEs, was associated with longer OS and PFS. We performed landmark analyses for OS and PFS to avoid lead-time bias due to the time-dependent nature of irAEs and to capture all patients other than those who experienced rapid progression, and our results were consistent with those of previous studies (20–25). These analyses revealed that unlike high-grade irAEs, low-grade irAEs were associated with a favorable prognosis.

A relationship between the irAE type and prognosis has been reported previously (24–34, 36, 37). A meta-analysis of patients with solid tumors showed that the patients with endocrine, skin, or gastrointestinal irAEs had longer OS (36). In addition, relatively small studies found that patients who developed skin disorders (24, 26–29), thyroiditis (25, 27, 30–32), and endocrine disorders (24) had a better prognosis, whereas those who developed pneumonitis (27, 33, 34) requiring cessation or interruption of ICI treatment and changes in treatment regimens had worse prognosis, consistent with our findings.

Notably, in this study, we found that low-grade non-skin/endocrine irAEs were associated with longer OS, whereas high-grade irAEs were associated with shorter OS. Although skin and endocrine irAEs can often be managed with the use of topical agents or hormone replacement therapy alone, respectively, the management of irAEs other than skin and endocrine disorders is difficult and often requires treatment with steroids and/or immunosuppressive drugs (42, 43). Although the cause of worse prognosis associated with severe irAEs is unclear, several reasons can be considered. As previously reported, 622 patients in this study had a survival event, with 12 patients (1.9%) dying from treatment-related AEs, first (39). In addition, the discontinuation of ICIs or possible reduced effectiveness of ICIs due to high-dose steroid usage, and the adverse effects of steroid administration could lead to poor prognosis, although some studies have found that steroid use did not affect the effectiveness of ICIs (44). Moreover, the occurrence of severe irAEs may negatively affect PFS and OS due to interruption of ICI therapy. Because the association between the types of irAEs and the effectiveness of ICIs has not been well studied, this study which observed 1,000 patients over a relatively long period provides important insights. Together, these findings suggest the need to develop treatment strategies for irAEs that do not negatively affect the effectiveness of ICIs.

In the J-TAIL study, approximately 20% of patients had prior ICI treatment (39), and it is assumed that some of these patients discontinued treatment due to irAEs. Patients who have experienced irAEs during ICI treatment are at risk of experiencing severe adverse reactions if they are re-exposed (45). Populations that have discontinued ICI treatment in the past due to the development of irAEs may have recurrent or newly developed irAEs during atezolizumab treatment, whereas response rates and PFS outcomes are not favorable in patients previously treated with ICIs (39), which may undermine the degree of association between irAEs and effectiveness.

Additionally, we performed an exploratory analysis of the association between the occurrence of irAEs and factors reported to be predictors of ICI effectiveness, including PD-L1 expression, tumor volume, CRP level, and the NLR (9–14). This study showed that the NLR and tumor volume were lower in patients who developed irAEs, and the association between PD-L1 expression and irAEs was unclear. It is likely that the patients who responded to atezolizumab received it for a longer period, resulting in a higher proportion of patients developing irAEs. For tumor volume, we expected that a larger tumor diameter would result in a stronger immune response and an increase in irAEs (46–48), but no such trend was observed. The CRP level, which is considered important in the management of irAEs (42, 43), was higher in patients who developed irAEs within the first 4 or 6 weeks of treatment than in those who did not develop irAEs. These trends were consistent, irrespective of the type of irAE. Because data on the relationship between the occurrence of irAEs and clinical factors are limited, the results of this study provide important insights into the management of irAEs.

This study has a few limitations. First, owing to its observational nature, the measurement of progression was prone to error, and the frequency of radiographic examinations for PFS evaluation varied across participating sites. Furthermore, as these analyses were of an exploratory nature, comparability between groups was not ensured when comparing subgroups, and multiplicity was not considered. Second, the median observation period was 11.5 months, which was insufficient to evaluate the “long-tail effect.” Third, the low incidence of some irAE subtypes limited the ability to assess their relationship with clinical benefits. Fourth, because this study was conducted in a real-world clinical setting and was not a clinical trial, some mild AEs, especially skin disorders, may not have been reported. The incidence of low-grade skin disorders was lower than that in a previous clinical trial (3). In addition, the assessment of irAEs was determined by the individual investigators. However, it is important to note that in Japan, there is widespread education on irAEs and established guidelines, which likely ensures appropriate and standardized differentiation and diagnosis. Fifth, this study was conducted only in Japan, and the results are based on the Japanese population; hence, the generalizability may be limited. Despite these limitations, this was one of the largest studies to examine the association between irAEs and the therapeutic effect of ICIs and included 74 patients who developed irAEs other than skin and endocrine disorders, for which limited data are available.

In conclusion, the occurrence of irAEs was associated with clinical benefit in patients with unresectable advanced or recurrent NSCLC treated with atezolizumab in a real-world setting. However, patients who developed grade ≥3 irAEs or other than skin and endocrine irAEs, tended to have shorter survival rates. The current findings suggest that to maximize the therapeutic effects of ICIs, it is necessary to improve the prognosis of patients with severe irAEs or other than skin and endocrine irAEs. There is an urgent need to develop treatments and management strategies for severe irAEs, particularly other than skin and endocrine irAEs.

## Supplementary Material

Supplementary Table S1 Supplementary Table S1. Background demographic and clinical characteristics of patients with or without irAE Abbreviations: ECOG PS, Eastern Cooperative Oncology Group performance status; EGFR, epidermal growth factor receptor; ICIs, immune checkpoint inhibitors; IHC, immunohistochemical staining; irAE, immune-related adverse event; PD-L1, programmed death ligand-1; TPS, tumor proportion score. a Data were missing for one patient. b Other includes patients with pleural effusion. c Negative, all targSupplementary Table driver oncogene statuses (EGFR mutation status, ALK rearrangement status, ROS1 rearrangement status, and BRAF V600E mutation status) were negative; positive, one or more were positive; unknown, none of the positives, any were unknown or not tested

Supplementary Table S2 Supplementary Table S2. Univariable and multivariable analysis of OS Abbreviations: ECOG PS, Eastern Cooperative Oncology Group performance status; HR, hazard ratio; ICI, immune checkpoint inhibitor; IHC, immunohistochemistry; irAE, immune-related adverse event; OS, overall survival; PD-L1, programmed death ligand-1; TPS, tumor proportion score.

Supplementary Table S3 Supplementary Table S3. Univariable and multivariable analysis of PFS Abbreviations: ECOG PS, Eastern Cooperative Oncology Group performance status; HR, hazard ratio; ICI, immune checkpoint inhibitor; IHC, immunohistochemistry; irAE, immune-related adverse event; PD-L1, programmed death ligand-1; PFS, progression-free survival; TPS, tumor proportion score.

Supplementary Table S4 Supplementary Table S4. Association between grade 1–2 irAEs and predictors of ICI effect a Mann–Whitney U Test. b Patients who had progression within 4 or 6 weeks after the initiation of treatment were excluded. Abbreviations: CRP, C-reactive protein; ICI, immune checkpoint inhibitor; IHC, immunohistochemical; irAE, immune-related adverse event; NLR, neutrophil-to-lymphocyte ratio; PD-L1, programmed death ligand-1; Q, quartile; SD, standard deviation.

Supplementary Table S5 Supplementary Table S5. Association between grade ≥3 irAEs and predictors of ICI effect a Mann–Whitney U Test. b Patients who had progression within 4 or 6 weeks after the initiation of treatment were excluded. Abbreviations: CRP, C-reactive protein; ICI, immune checkpoint inhibitor; IHC, immunohistochemical; irAE, immune-related adverse event; NLR, neutrophil-to-lymphocyte ratio; PD-L1, programmed death ligand-1; Q, quartile; SD, standard deviation.

Supplementary Table S6 Supplementary Table S6. Association between skin or endocrine disorder irAEs and predictors of ICI effect a Mann–Whitney U Test. b Patients who had progression within 4 or 6 weeks after the initiation of treatment were excluded. Abbreviations: CRP, C-reactive protein; ICI, immune checkpoint inhibitor; IHC, immunohistochemical; irAE, immune-related adverse event; NLR, neutrophil-to-lymphocyte ratio; PD-L1, programmed death ligand-1; Q, quartile; SD, standard deviation.

Supplementary Figure S1 Supplementary Figure S1. PFS according to the grade of irAE (A) 4 weeks landmark PFS of irAE according to the grade (B) 8 weeks landmark PFS of irAE according to the grade One event with undetermined grade was excluded from the analysis. Abbreviations: CI, confidence interval; HR, hazard ratio; irAE, immune-related adverse event; PFS, progression-free survival

Supplementary Figure S2 Supplementary Figure S2. PFS of irAE of skin or endocrine disorders (A) PFS of irAE of skin or endocrine disorders according to the onset (B) PFS of irAE of skin or endocrine disorders according to the grade One event with undetermined grade was excluded from the analysis according to grade. Abbreviations: CI, confidence interval; HR, hazard ratio; irAE, immune-related adverse event; PFS, progression-free survival 

Supplementary Figure S3 Supplementary Figure S3. PFS of irAE other than skin or endocrine disorders (A) PFS of irAE other than skin or endocrine disorders according to the onset (B) PFS of irAE other than skin or endocrine disorders according to the grade One event with undetermined grade was excluded from the analysis according to grade. Abbreviations: CI, confidence interval; HR, hazard ratio; irAE, immune-related adverse event; PFS, progression-free survival

Supplementary Figure S4 Supplementary Figure S4. OS according to number of whole irAEs Abbreviations: CI, confidence interval; HR, hazard ratio; irAE, immune-related adverse event; NE, not evaluable; NR, not reached; OS, overall survival
