# Supplementary material for: Association between Immune-Related Adverse Events and Atezolizumab in Previously Treated Patients with Unresectable Advanced or Recurrent Non–Small Cell Lung Cancer
Source: Cancer Res Commun. 2024 Nov 1;4(11):2858–67. doi: 10.1158/2767-9764.CRC-24-0212 (PMC11528261; doi:10.1158/2767-9764.CRC-24-0212)
Supplement: Supplementary Table S1 — Background demographic and clinical characteristics of patients with or without irAE Abbreviations: ECOG PS, Eastern Cooperative Oncology Group performance status; EGFR, epidermal growth factor receptor; ICIs, immune checkpoint inhibitors; IHC, immunohistochemical staining; irAE, immune-related adverse event; PD-L1, programmed death ligand-1; TPS, tumor proportion score. a Data were missing for one patient. b Other includes patients with pleural effusion. c Negative, all targSupplementary Table driver oncogene statuses (EGFR mutation status, ALK rearrangement status, ROS1 rearrangement status, and BRAF V600E mutation status) were negative; positive, one or more were positive; unknown, none of the positives, any were unknown or not tested [file crc-24-0212_supplementary_table_s1_suppst1.pdf]

Supplementary Table S1. Background demographic and clinical characteristics of patients with or without irAE

Characteristics	Safety analysis set N = 1002	With irAE N = 190	Without irAE N = 812
Sex			
Male	719 (71.8)	140 (73.7)	579 (71.3)
Female	283 (28.2)	50 (26.3)	233 (28.7)
Age, years			
Median age (range)	71 (34–93)	72 (44–93)	70 (34–91)
≥75	291 (29.0)	58 (30.5)	233 (28.7)
Histology[a]			
Squamous	216 (21.6)	39 (20.5)	177 (21.8)
Non-squamous	738 (73.7)	137 (72.1)	601 (74.0)
Other	47 (4.7)	14 (7.4)	33 (4.1)
ECOG PS[a]			
0	335 (33.4)	69 (36.3)	266 (32.8)
1	546 (54.5)	106 (55.8)	440 (54.2)
2	107 (10.7)	12 (6.3)	95 (11.7)
3	13 (1.3)	3 (1.6)	10 (1.2)
4	0 (0)	0 (0)	0 (0)
Smoking history[a]	756 (75.4)	153 (80.5)	603 (74.3)
Medical history[a]			
Autoimmune disease	20 (2.0)	7 (3.7)	13 (1.6)
Other than autoimmune disease	537 (53.6)	100 (52.6)	437 (53.8)
Complications[a]			
Autoimmune disease	68 (6.8)	21 (11.1)	47 (5.8)
Other than autoimmune disease	709 (70.8)	129 (67.9)	580 (71.4)
Primary tumor surgery[a]	287 (28.6)	54 (28.4)	233 (28.7)
Metastases[a]			
Brain	189 (18.9)	31 (16.3)	158 (19.5)
Bone	264 (26.3)	37 (19.5)	227 (28.0)
Adrenal	91 (9.1)	18 (9.5)	73 (9.0)
Liver	126 (12.6)	22 (11.6)	104 (12.8)
Kidney	13 (1.3)	4 (2.1)	9 (1.1)
Other[b]	806 (80.4)	156 (82.1)	650 (80.0)
Stage[a]			
IIIA	42 (4.2)	9 (4.7)	33 (4.1)
IIIB	60 (6.0)	15 (7.9)	45 (5.5)
IIIC	14 (1.4)	1 (0.5)	13 (1.6)
IVA	284 (28.3)	60 (31.6)	224 (27.6)
IVB	311 (31.0)	48 (25.3)	263 (32.4)
Post-surgery recurrence	212 (21.2)	47 (24.7)	165 (20.3)
Post chemoradiation therapy recurrence	78 (7.8)	10 (5.3)	68 (8.4)
Treatment line of atezolizumab			
1	1 (0.1)	0 (0)	1 (0.1)
2	425 (42.4)	99 (52.1)	326 (40.1)
3	221 (22.1)	31 (16.3)	190 (23.4)
≥4	355 (35.4)	60 (31.6)	295 (36.3)
Prior drug therapy[a]			
Immune checkpoint inhibitors	219 (21.9)	34 (17.9)	185 (22.8)
Chemotherapy	989 (98.7)	187 (98.4)	802 (98.8)
Angiogenesis inhibitor	388 (38.7)	54 (28.4)	334 (41.1)
EGFR inhibitor	156 (15.6)	18 (9.5)	138 (17.0)
ALK inhibitor	7 (0.7)	0 (0)	7 (0.9)
Other	7 (0.7)	1 (0.5)	6 (0.7)
Prior radiation therapy	288 (28.7)	54 (28.4)	234(28.8)
Targetable driver oncogene status[a]			
Negative[c]	128 (12.8)	27 (14.2)	101 (12.4)
Positive[c]	155 (15.5)	17 (8.9)	138 (17.0)

Characteristics	Safety analysis set N = 1002	With irAE N = 190	Without irAE N = 812
Unknown[c]	594 (59.3)	120 (63.2)	474 (58.4)
EGFR mutation status			
Positive	146 (14.6)	16 (8.4)	130 (16.0)
ALK rearrangement status			
Positive	5 (0.5)	0 (0)	5 (0.6)
ROS1 rearrangement status			
Positive	3 (0.3)	0 (0)	3 (0.4)
BRAF V600E mutation status			
Positive	1 (0.1)	1 (0.5)	0 (0)
PD-L1			
IHC 22C3			
N	802	152	650
TPS ≥50%	138 (17.2)	26 (17.1)	112 (17.2)
TPS 1–49%	308 (38.4)	57 (37.5)	251 (38.6)
TPS <1%	356 (44.4)	69 (45.4)	287 (44.2)

Abbreviations: ECOG PS, Eastern Cooperative Oncology Group performance status; EGFR, epidermal growth factor receptor; ICIs, immune checkpoint inhibitors; IHC, immunohistochemical staining; irAE, immune-related adverse event; PD-L1, programmed death ligand-1; TPS, tumor proportion score.

[a] Data were missing for one patient.

[b] Other includes patients with pleural effusion.

[c] Negative, all targSupplementary Table driver oncogene statuses (*EGFR* mutation status, *ALK* rearrangement status, *ROS1* rearrangement status, and *BRAF* V600E mutation status) were negative; positive, one or more were positive; unknown, none of the positives, any were unknown or not tested.
