# Supplementary material for: Association between Immune-Related Adverse Events and Atezolizumab in Previously Treated Patients with Unresectable Advanced or Recurrent Non–Small Cell Lung Cancer
Source: Cancer Res Commun. 2024 Nov 1;4(11):2858–67. doi: 10.1158/2767-9764.CRC-24-0212 (PMC11528261; doi:10.1158/2767-9764.CRC-24-0212)
Supplement: Supplementary Table S2 — Univariable and multivariable analysis of OS Abbreviations: ECOG PS, Eastern Cooperative Oncology Group performance status; HR, hazard ratio; ICI, immune checkpoint inhibitor; IHC, immunohistochemistry; irAE, immune-related adverse event; OS, overall survival; PD-L1, programmed death ligand-1; TPS, tumor proportion score. [file crc-24-0212_supplementary_table_s2_suppst2.pdf]

Supplementary Table S2. Univariable and multivariable analysis of OS

		Univariable		Multivariable	
**Variable**		**HR**	**P-value**	**Adjusted HR**	**P-value**
Sex	Male				
	Female	0.86 (0.72–1.03)	0.104	0.94 (0.75–1.18)	0.575
Age	<75 years				
	≥75 years	1.14 (0.96–1.35)	0.137	0.98 (0.78–1.23)	0.849
ECOG PS	0–1				
	≥2	3.50 (2.82–4.36)	<0.001	3.87 (2.92–5.13)	<0.001
Targetable driver oncogene status	Negative				
	Positive	1.08 (0.85–1.38)	0.537	1.19 (0.90–1.58)	0.227
Previous treatment with ICIs	No				
	Yes	1.42 (1.19–1.71)	<0.001	1.59 (1.24–2.06)	<0.001
PD-L1 (IHC 22C3)	TPS ≥1%				
	TPS <1%	0.84 (0.71–1.01)	0.060	0.87 (0.70–1.08)	0.208
Onset of irAEs	No				
	Yes	0.66 (0.54–0.82)	<0.001	0.71 (0.54–0.93)	0.012

Abbreviations: ECOG PS, Eastern Cooperative Oncology Group performance status; HR, hazard ratio; ICI, immune checkpoint inhibitor; IHC, immunohistochemistry; irAE, immune-related adverse event; OS, overall survival; PD-L1, programmed death ligand-1; TPS, tumor proportion score.
