# Supplementary material for: Association between Immune-Related Adverse Events and Atezolizumab in Previously Treated Patients with Unresectable Advanced or Recurrent Non–Small Cell Lung Cancer
Source: Cancer Res Commun. 2024 Nov 1;4(11):2858–67. doi: 10.1158/2767-9764.CRC-24-0212 (PMC11528261; doi:10.1158/2767-9764.CRC-24-0212)
Supplement: Supplementary Table S3 — Univariable and multivariable analysis of PFS Abbreviations: ECOG PS, Eastern Cooperative Oncology Group performance status; HR, hazard ratio; ICI, immune checkpoint inhibitor; IHC, immunohistochemistry; irAE, immune-related adverse event; PD-L1, programmed death ligand-1; PFS, progression-free survival; TPS, tumor proportion score. [file crc-24-0212_supplementary_table_s3_suppst3.pdf]

Supplementary Table S3. Univariable and multivariable analysis of PFS

Variable		Univariable		Multivariable	
		HR	P-value	Adjusted HR	P-value
Sex	Male				
	Female	1.14 (0.98–1.31)	0.082	1.13 (0.94–1.36)	0.198
Age	<75 years				
	≥75 years	0.92 (0.80–1.06)	0.247	0.90 (0.75–1.09)	0.293
ECOG PS	0–1				
	≥2	1.94 (1.60–2.36)	<0.001	1.76 (1.37–2.27)	<0.001
Targetable driver oncogene status	Negative				
	Positive	1.50 (1.24–1.82)	<0.001	1.51 (1.20–1.90)	<0.001
Previous treatment with ICIs	No				
	Yes	1.31 (1.12–1.53)	<0.001	1.38 (1.11–1.71)	0.003
PD-L1 (IHC 22C3)	TPS ≥1%				
	TPS <1%	1.06 (0.92–1.23)	0.401	1.10 (0.93–1.31)	0.272
Onset of irAEs	No				
	Yes	0.63 (0.53–0.75)	<0.001	0.66 (0.53–0.82)	<0.001

Abbreviations: ECOG PS, Eastern Cooperative Oncology Group performance status; HR, hazard ratio; ICI, immune checkpoint inhibitor; IHC, immunohistochemistry; irAE, immune-related adverse event; PD-L1, programmed death ligand-1; PFS, progression-free survival; TPS, tumor proportion score.
