# Supplementary material for: Association between Immune-Related Adverse Events and Atezolizumab in Previously Treated Patients with Unresectable Advanced or Recurrent Non–Small Cell Lung Cancer
Source: Cancer Res Commun. 2024 Nov 1;4(11):2858–67. doi: 10.1158/2767-9764.CRC-24-0212 (PMC11528261; doi:10.1158/2767-9764.CRC-24-0212)
Supplement: Supplementary Table S4 — Association between grade 1–2 irAEs and predictors of ICI effect a Mann–Whitney U Test. b Patients who had progression within 4 or 6 weeks after the initiation of treatment were excluded. Abbreviations: CRP, C-reactive protein; ICI, immune checkpoint inhibitor; IHC, immunohistochemical; irAE, immune-related adverse event; NLR, neutrophil-to-lymphocyte ratio; PD-L1, programmed death ligand-1; Q, quartile; SD, standard deviation. [file crc-24-0212_supplementary_table_s4_suppst4.pdf]

Supplementary Table S4. Association between grade 1–2 irAEs and predictors of ICI effect

Characteristic		Overall		Within the first 4 weeks		Within the first 6 weeks	
		With<br>N = 105	Without<br>N = 812	With<br>N = 48	Without [b]<br>N = 681	With<br>N = 46	Without[b]<br>N = 596
PD-L1 IHC (22C3), %	N	53	412	23	341	26	298
	Mean ± SD	21.3 ± 30.9	23.7 ± 31.4	19.6 ± 26.0	23.2 ± 31.2	20.5 ± 27.2	23.1 ± 31.6
	Median	5.0	5.0	5.0	5.0	5.0	5.0
	Q1, Q3	0.0, 25.0	0.0, 40.0	0.0, 25.0	0.0, 40.0	1.0, 25.0	0.0, 35.0
	Min, Max	0, 95	0, 100	0, 80	0, 100	0, 80	0, 100
	P-value[a]	0.741		0.875		0.715	
NLR	N	103	784	48	658	46	574
	Mean ± SD	3.42 ± 2.37	5.36 ± 7.62	3.96 ± 2.66	4.51 ± 5.21	3.78 ± 2.63	4.30 ± 5.06
	Median	2.80	3.45	2.99	3.17	2.95	3.02
	Q1, Q3	1.98, 4.30	2.24, 5.59	2.37, 4.66	2.12, 4.99	2.25, 4.69	2.07, 4.78
	Min, Max	0.5, 14.2	0.6, 109.1	0.9, 14.2	0.6, 67.7	0.9, 14.2	0.6, 67.7
	P-value[a]	**0.002**		0.895		0.953	
CRP, mg/dL	N	105	793	48	665	46	582
	Mean ± SD	1.55 ± 2.67	2.05 ± 3.69	1.95 ± 2.56	1.53 ± 2.82	1.77 ± 2.58	1.38 ± 2.54
	Median	0.52	0.58	0.98	0.42	0.78	0.38
	Q1, Q3	0.11, 1.80	0.15, 2.11	0.29, 2.88	0.13, 1.60	0.23, 1.86	0.12, 1.41
	Min, Max	0.0, 17.3	0.0, 39.4	0.0, 10.9	0.0, 25.5	0.0, 10.9	0.0, 22.9
	P-value[a]	0.362		**0.017**		0.066	
Tumor volume, mm	N	93	666	44	555	42	483
	Mean ± SD	45.11 ± 24.99	56.54 ± 36.98	50.02 ± 28.50	53.69 ± 34.32	45.27 ± 25.43	52.38 ± 33.99
	Median	40.30	47.00	44.90	45.00	41.00	44.00
	Q1, Q3	28.20, 54.80	30.00, 74.10	30.50, 61.40	29.00, 70.10	27.00, 54.00	28.00, 69.00
	Min, Max	10.0, 148.0	10.0, 244.4	10.0, 148.0	10.0, 244.4	10.0, 125.0	10.0, 244.4
	P-value[a]	**0.012**		0.754		0.299	

[a] Mann–Whitney *U* Test. [b] Patients who had progression within 4 or 6 weeks after the initiation of treatment were excluded.

Abbreviations: CRP, C-reactive protein; ICI, immune checkpoint inhibitor; IHC, immunohistochemical; irAE, immune-related adverse event; NLR, neutrophil-to-lymphocyte ratio; PD-L1, programmed death ligand-1; Q, quartile; SD, standard deviation.
