# Supplementary material for: Association between Immune-Related Adverse Events and Atezolizumab in Previously Treated Patients with Unresectable Advanced or Recurrent Non–Small Cell Lung Cancer
Source: Cancer Res Commun. 2024 Nov 1;4(11):2858–67. doi: 10.1158/2767-9764.CRC-24-0212 (PMC11528261; doi:10.1158/2767-9764.CRC-24-0212)
Supplement: Supplementary Table S5 — Association between grade ≥3 irAEs and predictors of ICI effect a Mann–Whitney U Test. b Patients who had progression within 4 or 6 weeks after the initiation of treatment were excluded. Abbreviations: CRP, C-reactive protein; ICI, immune checkpoint inhibitor; IHC, immunohistochemical; irAE, immune-related adverse event; NLR, neutrophil-to-lymphocyte ratio; PD-L1, programmed death ligand-1; Q, quartile; SD, standard deviation. [file crc-24-0212_supplementary_table_s5_suppst5.pdf]

## Supplementary Table S5. Association between grade ≥3 irAEs and predictors of ICI effect

| Characteristic | | Overall | | Within the first 4 weeks | | Within the first 6 weeks | |
|---|---|---|---|---|---|---|---|
| | | With N = 84 | Without N = 812 | With N = 34 | Without[b] N = 681 | With N = 31 | Without[b] N = 596 |
| PD-L1 IHC (22C3), % | N | 32 | 412 | 16 | 341 | 13 | 298 |
| | Mean ± SD | 24.9 ± 34.0 | 23.7 ± 31.4 | 24.7 ± 34.5 | 23.2 ± 31.2 | 22.7 ± 32.6 | 23.1 ± 31.6 |
| | Median | 5.0 | 5.0 | 5.0 | 5.0 | 5.0 | 5.0 |
| | Q1, Q3 | 0.0, 45.0 | 0.0, 40.0 | 0.5, 35.0 | 0.0, 40.0 | 0.0, 30.0 | 0.0, 35.0 |
| | Min, Max | 0, 90 | 0, 100 | 0, 90 | 0, 100 | 0, 90 | 0, 100 |
| | P-value[a] | 0.901 | | 0.700 | | 0.939 | |
| NLR | N | 83 | 784 | 34 | 658 | 31 | 574 |
| | Mean ± SD | 4.91 ± 6.04 | 5.36 ± 7.62 | 3.97 ± 4.45 | 4.51 ± 5.21 | 3.36 ± 2.06 | 4.30 ± 5.06 |
| | Median | 3.20 | 3.45 | 2.72 | 3.17 | 2.58 | 3.02 |
| | Q1, Q3 | 2.15, 4.78 | 2.24, 5.59 | 1.98, 4.57 | 2.12, 4.99 | 1.92, 4.57 | 2.07, 4.78 |
| | Min, Max | 0.6, 33.0 | 0.6, 109.1 | 1.1, 26.6 | 0.6, 67.7 | 1.1, 8.8 | 0.6, 67.7 |
| | P-value[a] | 0.413 | | 0.253 | | 0.301 | |
| CRP, mg/dL | N | 83 | 793 | 33 | 665 | 31 | 582 |
| | Mean ± SD | 1.89 ± 3.57 | 2.05 ± 3.69 | 1.97 ± 2.97 | 1.53 ± 2.82 | 1.81 ± 2.68 | 1.38 ± 2.54 |
| | Median | 0.51 | 0.58 | 0.52 | 0.42 | 0.52 | 0.38 |
| | Q1, Q3 | 0.17, 1.90 | 0.15, 2.11 | 0.20, 1.90 | 0.13, 1.60 | 0.17, 1.90 | 0.12, 1.41 |
| | Min, Max | 0.0, 26.3 | 0.0, 39.4 | 0.0, 11.8 | 0.0, 25.5 | 0.0, 11.8 | 0.0, 22.9 |
| | P-value[a] | 0.806 | | 0.213 | | 0.125 | |
| Tumor volume, mm | N | 73 | 666 | 31 | 555 | 30 | 483 |
| | Mean ± SD | 53.70 ± 33.01 | 56.54 ± 36.98 | 59.67 ± 35.99 | 53.69 ± 34.32 | 53.89 ± 33.58 | 52.38 ± 33.99 |
| | Median | 45.00 | 47.00 | 47.00 | 45.00 | 44.97 | 44.00 |
| | Q1, Q3 | 32.00, 75.00 | 30.00, 74.10 | 34.00, 78.00 | 29.00, 70.10 | 34.00, 63.50 | 28.00, 69.00 |
| | Min, Max | 10.0, 161.0 | 10.0, 244.4 | 10.0, 149.0 | 10.0, 244.4 | 10.0, 149.0 | 10.0, 244.4 |
| | P-value[a] | 0.647 | | 0.302 | | 0.700 | |

[a] Mann–Whitney *U* Test. [b] Patients who had progression within 4 or 6 weeks after the initiation of treatment were excluded.

Abbreviations: CRP, C-reactive protein; ICI, immune checkpoint inhibitor; IHC, immunohistochemical; irAE, immune-related adverse event; NLR, neutrophil-to-lymphocyte ratio; PD-L1, programmed death ligand-1; Q, quartile; SD, standard deviation.
