# Supplementary material for: Association between Immune-Related Adverse Events and Atezolizumab in Previously Treated Patients with Unresectable Advanced or Recurrent Non–Small Cell Lung Cancer
Source: Cancer Res Commun. 2024 Nov 1;4(11):2858–67. doi: 10.1158/2767-9764.CRC-24-0212 (PMC11528261; doi:10.1158/2767-9764.CRC-24-0212)
Supplement: Supplementary Table S6 — Association between skin or endocrine disorder irAEs and predictors of ICI effect a Mann–Whitney U Test. b Patients who had progression within 4 or 6 weeks after the initiation of treatment were excluded. Abbreviations: CRP, C-reactive protein; ICI, immune checkpoint inhibitor; IHC, immunohistochemical; irAE, immune-related adverse event; NLR, neutrophil-to-lymphocyte ratio; PD-L1, programmed death ligand-1; Q, quartile; SD, standard deviation. [file crc-24-0212_supplementary_table_s6_suppst6.pdf]

Supplementary Table S6. Association between skin or endocrine disorder irAEs and predictors of ICI effect

Characteristic		Overall		Within the first 4 weeks		Within the first 6 weeks	
		With N = 74	Without N = 812	With N = 25	Without[b] N = 681	With N = 25	Without[b] N = 596
PD-L1 IHC (22C3), %	N	34	412	11	341	13	298
	Mean ± SD	14.4 ± 26.6	23.7 ± 31.4	20.5 ± 32.4	23.2 ± 31.2	20.5 ± 30.6	23.1 ± 31.6
	Median	3.5	5.0	5.0	5.0	5.0	5.0
	Q1, Q3	0.0, 10.0	0.0, 40.0	0.0, 45.0	0.0, 40.0	0.0, 40.0	0.0, 35.0
	Min, Max	0, 90	0, 100	0, 90	0, 100	0, 90	0, 100
	P-value[a]	0.108		0.706		0.883	
NLR	N	73	784	25	658	25	574
	Mean ± SD	3.47 ± 3.62	5.36 ± 7.62	3.71 ± 2.20	4.51 ± 5.21	3.23 ± 2.13	4.30 ± 5.06
	Median	2.60	3.45	3.00	3.17	2.72	3.02
	Q1, Q3	1.92, 3.50	2.24, 5.59	2.56, 4.69	2.12, 4.99	1.92, 3.66	2.07, 4.78
	Min, Max	0.5, 28.8	0.6, 109.1	1.2, 10.1	0.6, 67.7	0.9, 10.1	0.6, 67.7
	P-value[a]	**< 0.001**		0.884		0.228	
CRP, mg/dL	N	73	793	24	665	25	582
	Mean ± SD	0.97 ± 1.60	2.05 ± 3.69	1.68 ± 2.40	1.53 ± 2.82	1.17 ± 2.23	1.38 ± 2.54
	Median	0.42	0.58	0.94	0.42	0.43	0.38
	Q1, Q3	0.08, 1.13	0.15, 2.11	0.20, 2.88	0.13, 1.60	0.12, 0.99	0.12, 1.41
	Min, Max	0.0, 10.9	0.0, 39.4	0.0, 10.9	0.0, 25.5	0.0, 10.9	0.0, 22.9
	P-value[a]	**0.024**		0.315		0.964	
Tumor volume, mm	N	65	666	22	555	23	483
	Mean ± SD	43.37 ± 29.56	56.54 ± 36.98	55.96 ± 37.16	53.69 ± 34.32	41.59 ± 28.34	52.38 ± 33.99
	Median	38.00	47.00	45.00	45.00	38.00	44.00
	Q1, Q3	23.00, 53.00	30.00, 74.10	35.00, 66.50	29.00, 70.10	21.00, 45.00	28.00, 69.00
	Min, Max	10.0, 148.0	10.0, 244.4	10.0, 148.0	10.0, 244.4	10.0, 125.0	10.0, 244.4
	P-value[a]	**0.002**		0.793		0.089	

[a] Mann–Whitney *U* Test. [b] Patients who had progression within 4 or 6 weeks after the initiation of treatment were excluded.

Abbreviations: CRP, C-reactive protein; ICI, immune checkpoint inhibitor; IHC, immunohistochemical; irAE, immune-related adverse event; NLR, neutrophil-to-lymphocyte ratio; PD-L1, programmed death ligand-1; Q, quartile; SD, standard deviation.
