# Supplementary material for: Association between Immune-Related Adverse Events and Atezolizumab in Previously Treated Patients with Unresectable Advanced or Recurrent Non–Small Cell Lung Cancer
Source: Cancer Res Commun. 2024 Nov 1;4(11):2858–67. doi: 10.1158/2767-9764.CRC-24-0212 (PMC11528261; doi:10.1158/2767-9764.CRC-24-0212)
Supplement: Supplementary Figure S1 — PFS according to the grade of irAE (A) 4 weeks landmark PFS of irAE according to the grade (B) 8 weeks landmark PFS of irAE according to the grade One event with undetermined grade was excluded from the analysis. Abbreviations: CI, confidence interval; HR, hazard ratio; irAE, immune-related adverse event; PFS, progression-free survival [file crc-24-0212_supplementary_figure_s1_suppsf1.pdf]

(A)

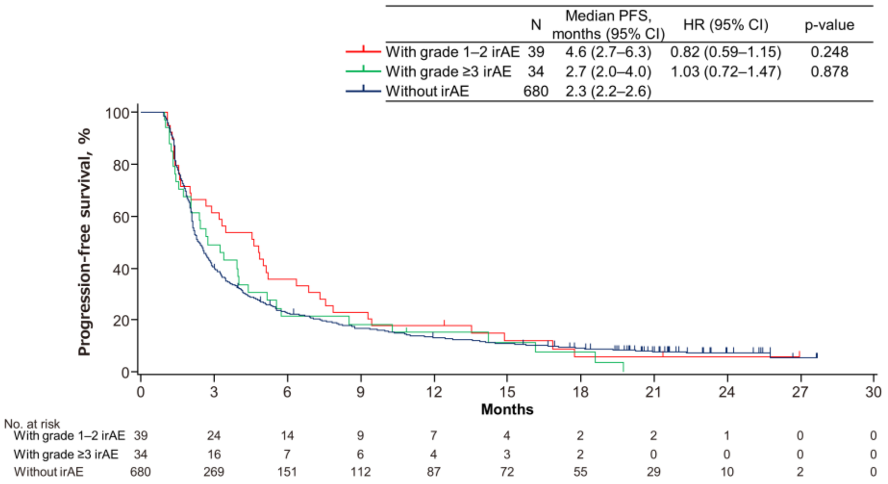

(B)

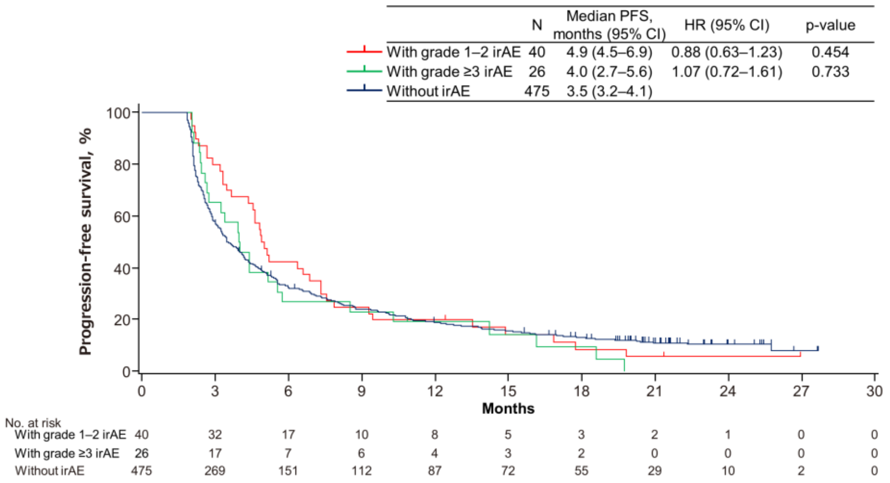

Supplementary Figure S1. PFS according to the grade of irAE

(A) 4 weeks landmark PFS of irAE according to the grade
(B) 8 weeks landmark PFS of irAE according to the grade
One event with undetermined grade was excluded from the analysis.
Abbreviations: CI, confidence interval; HR, hazard ratio; irAE, immune-related adverse event; PFS, progression-free survival
