# Supplementary material for: Association between Immune-Related Adverse Events and Atezolizumab in Previously Treated Patients with Unresectable Advanced or Recurrent Non–Small Cell Lung Cancer
Source: Cancer Res Commun. 2024 Nov 1;4(11):2858–67. doi: 10.1158/2767-9764.CRC-24-0212 (PMC11528261; doi:10.1158/2767-9764.CRC-24-0212)
Supplement: Supplementary Figure S3 — PFS of irAE other than skin or endocrine disorders (A) PFS of irAE other than skin or endocrine disorders according to the onset (B) PFS of irAE other than skin or endocrine disorders according to the grade One event with undetermined grade was excluded from the analysis according to grade. Abbreviations: CI, confidence interval; HR, hazard ratio; irAE, immune-related adverse event; PFS, progression-free survival [file crc-24-0212_supplementary_figure_s3_suppsf3.pdf]

(A)

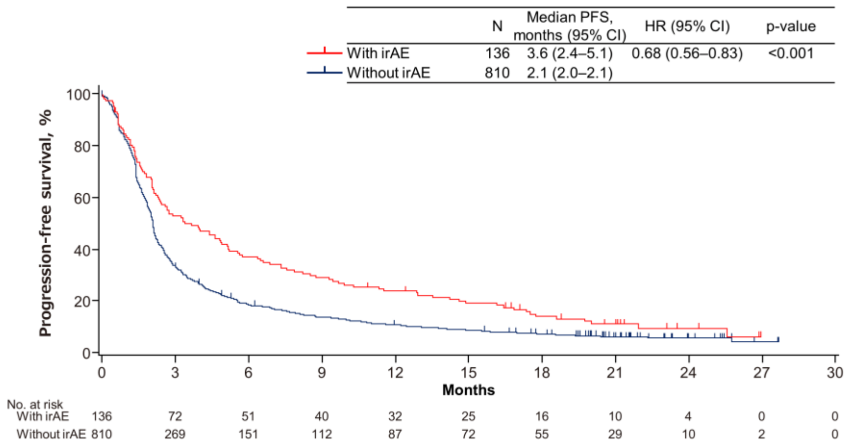

(B)

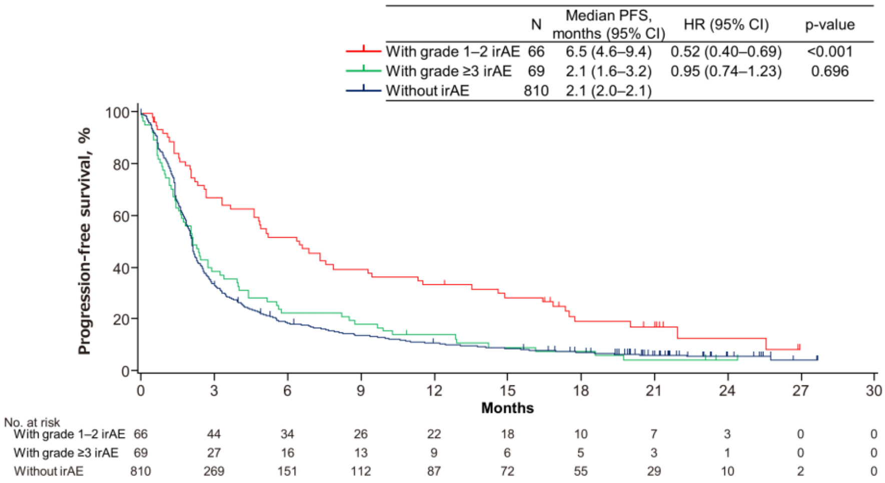

Supplementary Figure S3. PFS of irAE other than skin or endocrine disorders

(A) PFS of irAE other than skin or endocrine disorders according to the onset
(B) PFS of irAE other than skin or endocrine disorders according to the grade
One event with undetermined grade was excluded from the analysis according to grade.
Abbreviations: CI, confidence interval; HR, hazard ratio; irAE, immune-related adverse event; PFS, progression-free survival
