# Supplementary material for: Association between Immune-Related Adverse Events and Atezolizumab in Previously Treated Patients with Unresectable Advanced or Recurrent Non–Small Cell Lung Cancer
Source: Cancer Res Commun. 2024 Nov 1;4(11):2858–67. doi: 10.1158/2767-9764.CRC-24-0212 (PMC11528261; doi:10.1158/2767-9764.CRC-24-0212)
Supplement: Supplementary Figure S4 — OS according to number of whole irAEs Abbreviations: CI, confidence interval; HR, hazard ratio; irAE, immune-related adverse event; NE, not evaluable; NR, not reached; OS, overall survival [file crc-24-0212_supplementary_figure_s4_suppsf4.pdf]

	N	18-month OS rate, %	Median OS, months (95% CI)	HR (95% CI)	p-value
Multiple irAE	48	65.3	NR (18.3–NE)	0.58 (0.35–0.95)	0.031
Single irAE	142	48.7	16.4 (14.2–21.6)		

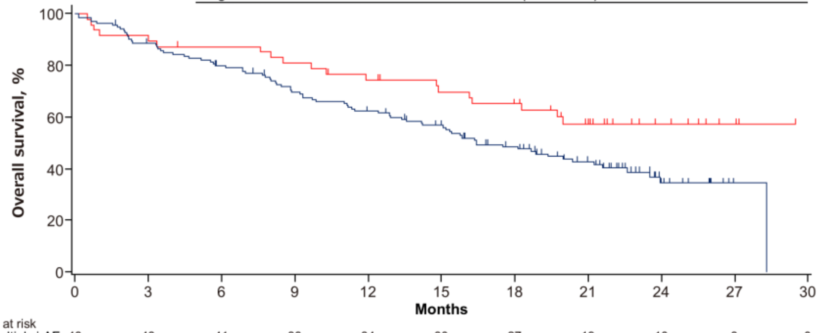

No. at risk

	0	3	6	9	12	15	18	21	24	27	30
Multiple irAE	48	43	41	38	34	30	27	19	10	3	0
Single irAE	142	124	110	94	82	70	55	38	11	1	0

Supplementary Figure S4. OS according to number of whole irAEs

Abbreviations: CI, confidence interval; HR, hazard ratio; irAE, immune-related adverse event; NE, not evaluable; NR, not reached; OS, overall survival
